# Analysis of risk factors for Gleason score upgrading after radical prostatectomy in a Chinese cohort

**DOI:** 10.1002/cam4.4274

**Published:** 2021-09-16

**Authors:** Baoling Zhang, Shangrong Wu, Yang Zhang, Mingyu Guo, Ranlu Liu

**Affiliations:** ^1^ Department of Urology The second hospital of Tianjin Medical University Tianjin China; ^2^ Tianjin Institute of Urology Tianjin China

**Keywords:** Gleason score, prostate biopsy, prostate cancer, radical prostatectomy

## Abstract

**Background:**

To study the risk factors of Gleason score upgrading (GSU) after radical prostatectomy (RP) in a Chinese cohort.

**Methods:**

The data of 637 patients who underwent prostate biopsy and RP in our hospital from January 2014 to January 2021 were retrospectively analyzed. The age, body mass index (BMI), prostate‐specific antigen (PSA) level, testosterone (TT) level, neutrophil‐to‐lymphocyte ratio (NLR), platelet‐to‐lymphocyte ratio (PLR), eosinophil‐to‐lymphocyte ratio (ELR), aspartate aminotransferase/alanine transaminase (AST/ALT) ratio, clinical stage, the biopsy method, and pathological characteristics of specimens after biopsy and RP were collected for all patients. Univariate analysis and multivariate logistic regression analysis were used to analyze the risk factors of GSU after RP. The predictive efficacy was verified with the area under the curve (AUC) of the receiver operating characteristic (ROC) curve. We performed the analysis separately in the overall cohort and in the cohort with Gleason score (GS) = 6.

**Results:**

In the overall cohort, 177 patients (27.79%) had GSU, and in the GS = 6 cohort, 68 patients (60.18%) had GSU. Multivariate logistic regression analysis showed that in the overall cohort, clinical stage ≥T2c (OR = 3.201, *p* < 0.001), the number of positive cores ≥3 (OR = 0.435, *p* = 0.04), and positive rate of biopsy (OR = 0.990, *p* = 0.016) can affect whether GS is upgraded, and the AUC of the combination of the three indicators for predicting the occurrence of GSU was 0.627. In the GS = 6 cohort, multivariate logistic regression analysis showed that clinical stage ≥T2c (OR = 4.690, *p* = 0.001) was a risk factor for GSU, and the AUC predicted to occur GSU is 0.675.

**Conclusion:**

Clinical stage ≥T2c, the number of positive cores <3, and lower positive rate of biopsy are the risk factors of GSU. This study may provide some references for clinicians to judge the accuracy of biopsy pathological grading and formulate treatment strategies, but the specific effect still needs clinical practice certification.

## Graphical abstract

Clinical stage ≥T2c, the number of positive cores <3, and lower positive rate of biopsy are the risk factors of Gleason score upgrading.

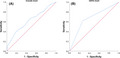

## 1 INTRODUCTION

Prostate cancer (PCa) is one of the most common malignant tumors in men around the world. Its incidence ranks first in American men, and its incidence in China is also increasing year by year.^1^ At present, the diagnosis of PCa mainly relies on prostate biopsy. However, even with the continuous progress of biopsy technology, there is still a difference in Gleason score (GS) between prostate biopsy and pathology after radical prostatectomy (RP).^2^, ^3^ The GS of biopsy is of great value in the diagnosis, grading, and prognosis of PCa. Therefore, it is of great significance to find out the relevant factors that can predict postoperative changes in GS to guide the clinical decision‐making.

The GS is applied to the grading of PCa, and the total score consists of the primary score and the secondary score, which classifies PCa into poorly differentiated, moderately differentiated, and well‐differentiated adenocarcinoma based on the total score. When Gleason score upgrading (GSU) occurs in PCa patients, the biochemical recurrence (BCR) rate increases, the local progression rate increases, and the cancer‐specific survival (CSS) decreases, which seriously affects the prognosis of patients.^4^ Fu et al.^5^ reported that low‐risk PCa patients with upgraded Gleason score had lower PSA recurrence‐free survival (*p* < 0.001), and patients with seminal vesicle invasion and extracapsular expansion were more likely to have GSU (both < 0.001). Santok et al.^6^ compared the 5‐year BCR survival rate, CSS, and overall survival (OS) of those with GSU from 6 to 7 and ≥8. It was found that the 5‐year BCR‐free survival rate, CSS, and OS of those with GSU to ≥8 were lower, and those with vascular lymphatic infiltration and nerve invasion were more likely to have GSU.

Although there are already some models to predict GSU,^7^, ^8^ there is evidence that the risk of GSU is very different between ethnic groups.^9^ The epidemiological characteristics and patient spectrum of PCa in China and other Asian countries with similar conditions are quite different from those in the west,^10^, ^11^ which are manifested in the following aspects: First, because PSA screening in China is not as widespread as in Western and other countries, more patients are already at a higher GS when diagnosed; as shown in a study report on Asian population, 80% of patients had GS ≥7 at the time of diagnosis of PCa.^11^ In this Chinese population‐based study, all included patients were studied according to the overall population and the population with GS = 6. Second, a controlled study of Caucasian and Asian populations showed that the incidence of high‐grade PCa in Asian men without screening was higher than in Caucasian men without screening, and the difference was still significant even after adjusting for prostate weight and age, which indicated that there was a difference in the Gleason scoring system between the two populations.^12^ Third, studies from Japan and South Korea indicated that the predictive role of models based on Western populations to predict GSU in Asian populations still has limitations.^12^, ^13^ Although some studies have been preliminarily explored based on Asian population, limited to the involved factors and sample size, the conclusions drawn from these studies need to be further verified.^7^, ^13^, ^14^, ^15^

Therefore, we conducted a retrospective study in a Chinese cohort to investigate the consistency between prostate biopsy and pathological grade after RP, and analyze the risk factors affecting GSU.

## 2 MATERIALS AND METHODS

The data of 637 patients with PCa who underwent RP in the second hospital of Tianjin Medical University from January 2014 to January 2021 were retrospectively collected, including age, body mass index (BMI), prostate‐specific antigen (PSA) level, testosterone (TT) level, neutrophil‐to‐lymphocyte ratio (NLR), platelet‐to‐lymphocyte ratio (PLR), eosinophil‐to‐lymphocyte ratio (ELR), aspartate aminotransferase/alanine transaminase (AST/ALT) ratio, clinical stage, the biopsy method, pathological characteristics of the specimens after biopsy and RP. The Institutional Review Committee and the Medical Ethics Committee of the Second Hospital of Tianjin Medical University approved the research protocol of this study, and the informed consent was obtained and signed by the patients and their families.

Inclusion criteria were as follows: (1) Prostate biopsy and RP must be performed in our hospital; (2) Endocrine therapy, chemotherapy, or radiotherapy was not performed before RP; and (3) Have complete clinical and pathological data. Exclusion criteria were as follows: (1) There was clinical evidence of inflammatory disease (such as infection) and (2) At the same time, they have diseases that cause abnormal liver function.

Prostate biopsy was performed jointly by two senior physicians. Before biopsy, multi‐parameter magnetic resonance imaging (mp‐MRI) was routinely performed, and some patients were given antibiotics to prevent infection. All patients underwent ultrasound‐guided transrectal or transperineal biopsy and RP using laparoscopic or robot‐assisted laparoscopic radical prostatectomy. Biopsy specimens and postoperative gross specimens pathological examination and diagnosis were jointly completed by the same two pathologists with senior professional title, and then they scored the GS according to the 2014 International Society of Urological Pathology (ISUP) Consensus Conference on Gleason Grading of Prostatic Carcinoma.^16^ We defined the GSU as: (1) the total score of GS of the specimen after RP was greater than that of the biopsy specimen and (2) GS changed from 3 + 4 at biopsy to 4 + 3 after RP.

SPSS 25.0 software was used to conduct statistical analysis on the data of the overall cohort and the cohort with GS = 6. The measurement data conforming to normal distribution are expressed as mean ± standard deviation (Mean ± SD), the measurement data conforming to skewed distribution were expressed as median (interquartile range, IQR), and the enumeration data were expressed as the number of cases and their percentages. Univariate analysis used independent sample *t‐*test, chi‐squared test, and rank sum test. Variables with statistical significance were entered into multivariate logistic regression analysis and the corresponding odds ratio and 95CI% were calculated. Generating a receiver operating characteristic curve (ROC) and calculating the area under the curve (AUC) to evaluate the predictive ability of statistically significant indicators. *p* < 0.05 was considered statistically significant.

## 3 RESULTS

### 3.1 Characteristics of the included population

A total of 637 patients were included. The clinical and pathological characteristics of the overall cohort and GS = 6 cohort are shown in Table 1. In the overall cohort, 177 (27.79%) patients had GSU, 301 (47.25%) patients had unchanged GS, and 159 (24.96%) patients had decreased GS; in the cohort with GS = 6, 68 (60.18%) patients had GSU and 45 (39.82%) patients had unchanged GS (Table 2).

**TABLE 1:** Clinical and pathological data of the patients

Variables	Overall cohort (*n* = 637)		GS = 6 cohort (*n* = 113)	
	No GSU (*n* = 460)	GSU (*n* = 177)	No GSU (*n* = 45)	GSU (*n* = 68)
Age (years), Mean(SD)	68.12 (6.74)	67.31 (7.21)	66.93 (6.75)	66.44 (6.61)
BMI (kg/m^2^), Mean(SD)	25.16 (3.89)	24.71 (3.85)	24.87 (2.50)	24.85 (2.92)
PSA (ng/ml), Median (IQR)	15.73 (20.96)	16.3 (37.83)	10.04 (7.32)	8.71 (7.41)
TT (ng/ml), Median (IQR)	4.24 (2.56)	4.13 (2.52)	4.28 (2.31)	4.16 (2.14)
NLR, Median (IQR)	2.19 (1.20)	2.12 (1.30)	2.35 (0.93)	2.01 (1.50)
PLR, Median (IQR)	116.58 (53.84)	121.28 (63.71)	120 (57.98)	120.83 (75.13)
ELR, Median (IQR)	0.064 (0.079)	0.064 (0.075)	0.065 (0.101)	0.055 (0.066)
AST/ALT ratio, Median (IQR)	0.99 (0.43)	1.03 (0.35)	0.92 (0.40)	1.05 (0.38)
Clinical stage, No (%)				
T1c	30 (6.5%)	9 (5.1%)	17 (37.8%)	9 (13.2%)
T2a	47 (10.2%)	18 (10.2%)	11 (24.4%)	12 (17.6%)
T2b	49 (10.7%)	8 (4.5%)	5 (11.1%)	5 (7.4%)
T2c	119 (25.9%)	31 (17.5%)	11 (24.4%)	18 (26.5%)
T3a	119 (25.9%)	67 (37.9%)	1 (2.2%)	20 (29.4%)
T3b	84 (18.3%)	38 (21.5%)	0	4 (5.9%)
T4	12 (2.6%)	6 (3.4%)	0	0
Method of diagnosis, No (%)				
TRUS	73 (15.9%)	34 (19.2%)	4 (8.9%)	10 (14.7%)
Transperineal biopsy	387 (84.1%)	143 (80.8%)	41 (91.1%)	58 (85.3%)
Biopsy specimens features				
Biopsy cores, Median (IQR)	24 (14.00)	22 (13.50)	30 (11.00)	24 (14.00)
Positive cores, Median (IQR)	7 (7.00)	5 (8.00)	2 (3.00)	3 (3.00)
% of positive cores, Median (IQR)	36.52 (41.67)	25 (40.00)	8.33 (10.96)	10.91 (15.47)
Maximum percentage of cancer per core, Median (IQR)	80 (50.00)	70 (50.00)	30 (45.00)	50 (50.00)
Biopsy Gleason score, No (%)				
6	45 (9.8%)	68 (38.4%)	45 (100.0%)	68 (100.0%)
3+4	77 (16.7%)	38 (21.5%)	0	0
4+3	86 (18.7%)	24 (13.6%)	0	0
8	197 (42.8%)	46 (26.0%)	0	0
9	47 (10.2%)	1 (0.6%)	0	0
10	8 (1.7%)	0	0	0
RP specimens features				
RP Gleason score, No (%)				
6	56 (12.2%)	0	45 (100.0%)	0
3+4	129 (28.0%)	56 (31.6%)	0	56 (82.4%)
4+3	121 (26.3%)	32 (18.1%)	0	10 (14.7%)
8	113 (24.5%)	38 (21.4%)	0	2 (2.9%)
9	40 (8.7%)	50 (28.2%)	0	0
10	1 (0.2%)	1 (0.6%)	0	0
PSM, No (%)	165 (35.9%)	88 (49.7%)	2 (2.2%)	23 (33.8%)
SVI, No (%)	98 (21.3%)	43 (24.3%)	0	4 (5.9%)
EPE, No (%)	172 (37.4%)	91 (51.4%)	1 (2.2%)	20 (29.4%)
LNP, No (%)	25 (5.4%)	9 (5.1%)	0	0
Nerve, No (%)	153 (33.3%)	67 (37.9%)	2 (4.4%)	13 (19.1%)

Abbreviations: BMI, body mass index; ELR, eosinophil‐to lymphocyte ratio; EPE, extraprostatic extension; GS, Gleason score; GSU, Gleason upgrading; IQR, interquartile range; LMP, lymph node positive; NLR, neutrophil‐to‐lymphocyte ratio; PLR, platelet‐to‐lymphocyte ratio; PSA, prostate‐specific antigen; PSM, positive surgical margin; RP, radical prostatectomy; SD, standard deviation; SVI, seminal vesicle invasion; TRUS, transrectal ultrasound; TT, testosterone.

**TABLE 2:** Comparison of Gleason score between radical prostatectomy and prostate biopsy

Biopsy Gleason score	RP Gleason score						Total
	6	3 + 4	4 + 3	8	9	10	
6	45 (39.82%)	56 (49.56%)	10 (8.85%)	2 (1.77%)	0	0	113 (17.74%)
3 + 4	7 (6.09%)	70 (60.87%)	22 (19.13%)	15 (13.04%)	1 (0.87%)	0	115 (18.05%)
4 + 3	1 (0.91%)	35 (31.82%)	50 (45.45%)	21 (19.09%)	3 (2.73%)	0	110 (17.27%)
8	3 (1.23%)	24 (9.88%)	70 (28.81%)	100 (41.15%)	46 (18.93%)	0	243 (38.15%)
9	0	0	1 (2.08%)	11 (22.92%)	35 (72.92%)	1 (2.08%)	48 (7.54%)
10	0	0	0	2 (25%)	5 (62.5%)	1 (12.5%)	8 (1.26%)
Total	56 (8.79%)	185 (29.04%)	153 (24.02%)	151 (23.70%)	90 (14.13%)	2 (0.31%)	637 (100%)

Abbreviation: RP, radical prostatectomy.

### 3.2 Factors affecting GSU

**FIGURE 1:**
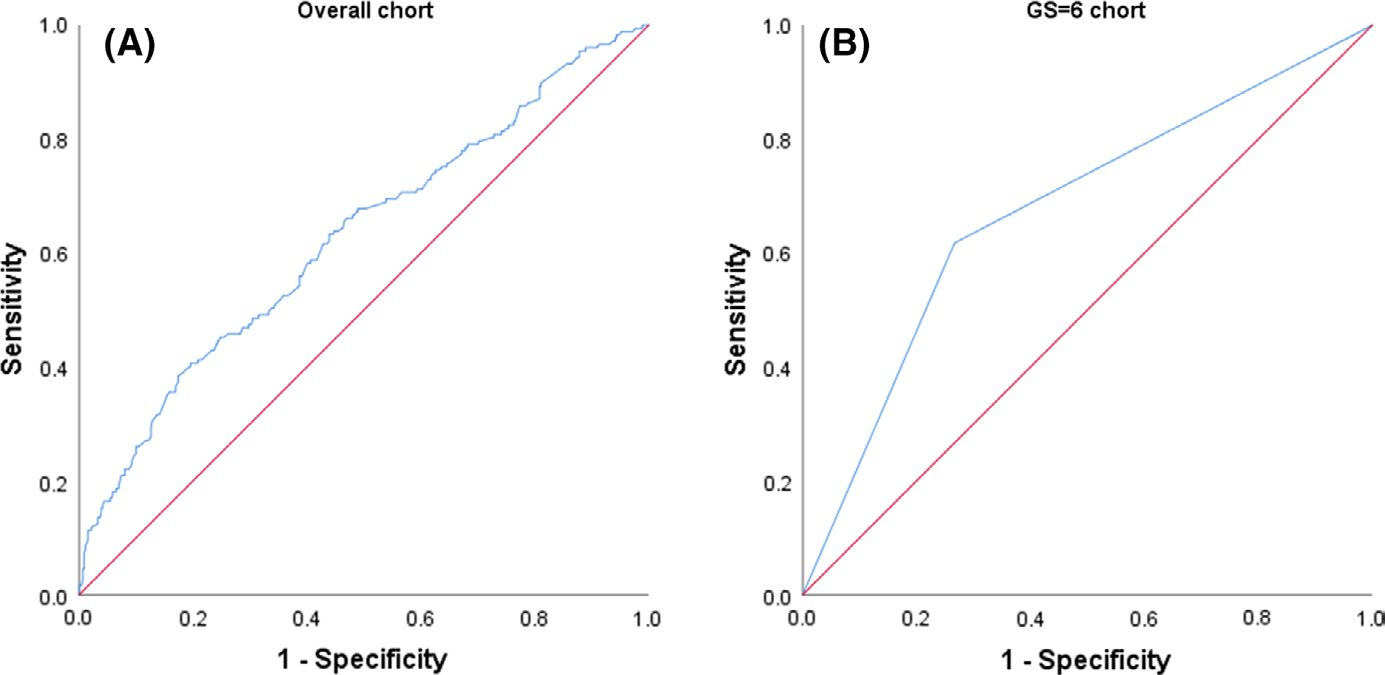
(A) Receiver operating characteristic (ROC) curves in predicting Gleason score upgrading (GSU) by clinical stage, the number of positive cores, and positive biopsy rate in the overall cohort. (B) ROC curves in predicting GSU by clinical stage in the GS = 6 cohort

The results of univariate analysis and multivariate logistic regression analysis in the overall cohort and GS = 6 cohort are shown in Table 3. In the overall cohort, the results of univariate analysis showed that clinical stage ≥T2c (*p* = 0.048), the number of positive cores ≥3 (*p* = 0.005), and the positive rate of biopsy (*p* = 0.004) were statistically significant for affecting whether the GS was upgraded, while the results of multivariate logistic regression analysis showed that clinical stage ≥T2c (*p* < 0.001) was a risk factor for GSU, while the number of positive cores ≥3 (*p* = 0.04) and higher positive rate of biopsy (*p* = 0.016) were protective factors for GSU. ROC curve analysis was performed for the efficacy of clinical stage, number of positive cores, and biopsy positive rate as possible influencing factors in predicting postoperative GSU, AUC = 0.627, *p* < 0.001, 95% CI: 0.577–0.677 (Figure 1A). In the GS = 6 cohort, the results of univariate analysis showed that both clinical stage ≥T2c (*p* < 0.001) and the positive rate of biopsy (*p* = 0.047) were statistically significant for affecting whether GSU occurred, while the results of multivariate logistic regression analysis showed that only clinical stage ≥T2c was a risk factor for GSU (*p* = 0.001). The AUC of clinical stage ≥T2c for predicting the occurrence of postoperative GSU was 0.675, *p* = 0.002, 95% CI: 0.574–0.777 (Figure 1B).

**TABLE 3:** Univariate analysis and multivariate logistic regression analysis of Gleason score upgrading after radical prostatectomy in overall cohort and GS = 6 cohort

Predictors	Overall cohort					GS = 6 cohort				
	Univariate analysis		Multiple logistic regression			Univariate analysis		Multiple logistic regression		
	t/c^2^ value	*p* value	OR	95%CI	*p* value	t/c^2^ value	*p* value	OR	95%CI	*p* value
Age	1.335	0.182				0.384	0.702			
BMI	1.305	0.192				0.035	0.972			
Non‐obesity (BMI < 28)	1.00 (Reference)					1.00 (Reference)				
Obesity (BMI > 28)	0.712	0.399				0.502	0.478			
PSA	—	0.265				—	0.697			
TT	—	0.388				—	0.740			
NLR										
<3	1.00 (Reference)					1.00 (Reference)				
≥3	0.694	0.405				0.032	0.858			
PLR	—	0.365				—	0.944			
ELR	—	0.813				—	0.995			
AST/ALT ratio	—	0.577				—	0.217			
Clinical stage										
≤T2b	1.00 (Reference)					1.00 (Reference)				
≥T2c	3.927	0.048	3.201	1.874–5.467	<0.001	13.370	<0.001	4.690	1.815–12.123	0.001
Method of diagnosis										
TRUS	1.00 (Reference)					1.00 (Reference)				
Transperineal biopsy	1.02	0.313				0.844	0.358			
Biopsy cores										
≤12	1.00 (Reference)					1.00 (Reference)				
>12	0.363	0.547				<0.001	0.988			
Positive cores										
<3	1.00 (Reference)					1.00 (Reference)				
≥3	7.791	0.005	0.435	0.246–0.767	0.04	1.818	0.178			
% of positive cores	—	0.004	0.990	0.982–0.998	0.016	—	0.047	0.996	0.958–1.034	0.819
Maximum percentage of cancer per core	—	0.674				—	0.055			

Abbreviations: BMI, body mass index; CI, confidence interval; ELR, eosinophil‐to‐lymphocyte ratio; GS, Gleason score; NLR, neutrophil‐to‐lymphocyte ratio; OR, odds ratio; PLR, platelet‐to‐lymphocyte ratio; PSA, prostate‐specific antigen; TRUS, transrectal ultrasound; TT, testosterone.

## 4 DISCUSSION

Prostate biopsy is the gold standard for the diagnosis of PCa, and a more accurate tumor grade can be established before surgery in combination with the results of PSA and mp‐MRI before biopsy.^17^, ^18^, ^19^, ^20^ Due to the limited tissue sampled for prostate biopsy, both transrectal and transperineal biopsies have different degrees of missed detection. It is reported in the literature that the positive rate of traditional biopsy for patients with suspected PCa is 26%. In recent years, magnetic resonance imaging fusion biopsy provides accurate navigation for prostate biopsy, and the positive rate of biopsy has been greatly improved. However, according to the literature reports, the consistency of biopsy GS and postoperative GS is about 50%.^3^, ^21^ Even in patients with PCa with positive single core biopsy, the incidence of postoperative GSU reached 34.9%.^22^

GS is the most important indicator to evaluate the biological characteristics of PCa, and it is one of the important reference factors for treatment and prognosis. The pathological GS after RP is closely related to biochemical recurrence (BCR) and disease‐free survival (DFS), so the correct evaluation of GS after RP is the key to treatment and prognosis.^23^, ^24^ In this study, we found that in the overall cohort, clinical stage ≥T2c was a risk factor for GSU, while the number of positive cores ≥3 and higher positive rate of biopsy were protective factors for GSU; in the cohort with GS = 6, only clinical stage ≥T2c was a risk factor for GSU.

The effect of clinical stage on GSU is still controversial, Jain et al.^25^ found that clinical stage T2 was an independent risk factor for GSU in a study of 862 PCa patients undergoing active surveillance. Moussa et al.^26^ found that clinical stage was a risk factor for GSU in a study of patients with biopsy GS of 6 and 7 (3+4), while Leyh‐Bannurah et al.^27^ did not find a correlation between clinical stage and GSU. In this study, we found that clinical stage >T2c was a risk factor for GSU in the overall cohort and in the GS = 6 cohort.

Truong et al.^28^ believe that the positive percentage of biopsy is related to postoperative GSU. The larger the total number of biopsy cores are, the greater the number of positive biopsy cores and the positive rate of biopsy are, which can relatively better reflect the overall condition of prostate and reduce the possibility of GSU due to the difference between local pathological condition and overall pathological condition. Serkin et al.^29^ reported that patients with positive biopsy percentage of ≤30% were 1.4 times more likely to have GSU than patients with positive biopsy percentage of 30%–50%, and higher than those with positive biopsy percentage >50% 2 times. Bandarage et al.^30^ found that taking biopsy positive rate <25% as the reference value 1, the odds ratio (OR) value for GSU with biopsy positive rate of 25%–65% was 0.7, and the OR value for GSU with biopsy positive rate of 65% was 0.6. These studies are consistent with our conclusion that the risk of GSU gradually decreases as the number of positive cores and the positive rate of biopsy increase. However, we also note that there are some studies that are contrary to our results, in which they concluded that the more the number of biopsy cores and the higher the biopsy positive rate, the greater the risk of GSU.^20^, ^31^, ^32^ We discussed this opposite view, and believed that the majority of prostate biopsies in this study were transperineal saturation biopsy (defined as the total number of biopsy cores ≥20), which accounted for 62.32% of all patients, so the number of biopsy‐positive cores ≥3 and the higher positive rate of biopsy accurately reflected the distribution of prostate tumor cells in the prostate, which also reduced the probability of GSU after RP. However, most of the studies that came to the opposite conclusion were traditional transrectal ultrasound‐guided prostate systematic biopsy, and the number of biopsy cores was mostly 12. If the number of biopsy‐positive cores was more and the positive rate of biopsy was higher with less total number of biopsy cores, it reflected that the number of tumor cells was more, so the probability of GSU may increase.

This study also has some limitations. First, this study was retrospective and prone to selection bias and recall bias. Second, limited to the completeness of clinical data, some indicators are not included in the statistics, such as prostate volume and PI‐RADS score of mp‐MRI, and these indicators have been confirmed to have an effect on GSU.^33^, ^34^, ^35^, ^36^ Third, this study is a single‐center study, and the study results still need multi‐center, large‐sample data for further validation.

## 5 CONCLUSION

Although some studies have investigated the risk factors of GSU in the Chinese cohort, our study included more clinical and pathological indicators and creatively included the potential influencing factor of the ratio of AST to ALT, because our previous studies found that it was different in patients with PCa and benign prostatic hyperplasia.^37^ In this study, final GSU occurred in 27.79% of patients in the overall cohort and 60.18% of patients in the cohort with GS = 6. In the overall cohort, clinical stage ≥T2c, number of positive cores < 3, and lower biopsy positive rate were risk factors for GSU; in the cohort with GS = 6, clinical stage ≥T2c was a risk factor for GSU. The results of this study can guide clinicians to assess the risk of GSU after RP and facilitate the development of more precise treatment plans for patients and the notification of the condition before RP. However, large sample size and multi‐center studies are still needed to verify this conclusion.

## CONFLICT OF INTEREST

All authors declare no conflict of interest.

## AUTHOR CONTRIBUTIONS

BZ and RL were involved in conception and design, revising it for intellectual content, and gave final approval of the completed article. SW, MG, and YZ were involved in extraction of data, and drafting the article. All authors read and approved the final manuscript.

## ETHICAL APPROVAL

All procedures performed in studies involving human participants were in accordance with the ethical standards of the institutional and/or national research committee and with the 1964 Helsinki declaration and its later amendments or comparable ethical standards.

## INFORMED CONSENT

Informed consent was obtained from all individual participants included in the study.

## CONSENT FOR PUBLICATION

All authors agree to publish this article.

## Data Availability

All data generated or analyzed during this study are included in this published article.
